# High throughput sequencing identifies an imprinted gene, Grb10, associated with the pluripotency state in nuclear transfer embryonic stem cells

**DOI:** 10.18632/oncotarget.17185

**Published:** 2017-04-18

**Authors:** Hui Li, Shuai Gao, Hua Huang, Wenqiang Liu, Huanwei Huang, Xiaoyu Liu, Yawei Gao, Rongrong Le, Xiaochen Kou, Yanhong Zhao, Zhaohui Kou, Jia Li, Hong Wang, Yu Zhang, Hailin Wang, Tao Cai, Qingyuan Sun, Shaorong Gao, Zhiming Han

**Affiliations:** ^1^ State Key Laboratory of Stem Cell and Reproductive Biology, Institute of Zoology, Chinese Academy of Sciences, Beijing, People's Republic of China; ^2^ University of Chinese Academy of Sciences, Chinese Academy of Science, Beijing, People's Republic of China; ^3^ National Institute of Biological Sciences, NIBS, Beijing, People's Republic of China; ^4^ Clinical and Translational Research Center of Shanghai First Maternity & Infant Hospital, School of Life Sciences and Technology, Tongji University, Shanghai, People's Republic of China; ^5^ State Key Laboratory of Environment Chemistry and Ecotoxicology, Research Center for Eco-Environmental Sciences, Chinese Academy of Science, Beijing, People's Republic of China

**Keywords:** nuclear transfer reprogramming, induced pluripotent reprogramming, Dlk1-Dio3 region, imprinted gene, RNA-seq

## Abstract

Somatic cell nuclear transfer and transcription factor mediated reprogramming are two widely used techniques for somatic cell reprogramming. Both fully reprogrammed nuclear transfer embryonic stem cells and induced pluripotent stem cells hold potential for regenerative medicine, and evaluation of the stem cell pluripotency state is crucial for these applications. Previous reports have shown that the Dlk1-Dio3 region is associated with pluripotency in induced pluripotent stem cells and the incomplete somatic cell reprogramming causes abnormally elevated levels of genomic 5-methylcytosine in induced pluripotent stem cells compared to nuclear transfer embryonic stem cells and embryonic stem cells. In this study, we compared pluripotency associated genes Rian and Gtl2 in the Dlk1-Dio3 region in exactly syngeneic nuclear transfer embryonic stem cells and induced pluripotent stem cells with same genomic insertion. We also assessed 5-methylcytosine and 5-hydroxymethylcytosine levels and performed high-throughput sequencing in these cells. Our results showed that Rian and Gtl2 in the Dlk1-Dio3 region related to pluripotency in induced pluripotent stem cells did not correlate with the genes in nuclear transfer embryonic stem cells, and no significant difference in 5-methylcytosine and 5-hydroxymethylcytosine levels were observed between fully and partially reprogrammed nuclear transfer embryonic stem cells and induced pluripotent stem cells. Through syngeneic comparison, our study identifies for the first time that Grb10 is associated with the pluripotency state in nuclear transfer embryonic stem cells.

## INTRODUCTION

Reprogramming refers to the erasure and remodeling of epigenetic marks during mammalian development *in vivo* and is an approach that changes differentiated cells into dedifferentiated cells *in vitro*. Somatic cell nuclear transfer (SCNT) and transcription factor (TF) mediated reprogramming are two major *in vitro* reprogramming techniques.

The studies of mammalian cloning and reprogram-ming have grown substantially since the first somatic cell cloned sheep, Dolly, was born [1]. The derivation of embryonic stem cells (ESCs) from cloned embryos by SCNT was an important achievement, and nuclear transfer ESCs (ntESCs) can be successfully derived from various adult cell types from mice, rhesus macaques, and humans, among others [2–7]. However, the reprogramming efficiency of SCNT limits the applications of ntESCs, although many solutions have been developed to resolve this issue. The addition of trichostatin A (TSA) and scriptaid (SCR) to the culture medium can improve SCNT efficiency [8–11]. *Xist*-deficient cumulus cells and Sertoli cells have been shown to robustly improve efficiency for mouse SCNT [12], and Kdm4A overexpression increased the blastocyst formation rate of human SCNT embryos [13].

Takahashi and Yamanaka demonstrated that pluripotent stem cells can be obtained from mouse embryonic or adult fibroblasts by introducing four transcription factors, Oct3/4, Sox2, c-Myc, and Klf4, under embryonic stem cell culture conditions [14]. Extensive studies examining TF mediated reprogramming were performed following the discovery that induced pluripotent stem cells (iPSCs) can support the full-term development of tetraploid blastocyst complemented embryos in mice [15, 16]. Many studies have been performed to improve efficiency. Small molecules such as Vitamin C have been used to improve efficiency both in mouse and human TF mediated pluripotent reprogramming [17]. A recent study found that combining several small molecules can reprogram mouse somatic cells, increasing reprogramming efficiency to 0.2% [18]. Moreover, the expression of certain genes can improve the TF mediated reprogramming efficiency. Zscan4 overexpression increased iPSCs efficiency and quality in mice [18], whereas Nr5a2 can replace Oct4 during reprogramming and improve efficiency in mice [19].

To better understand SCNT and TF mediated reprogramming, the methylation state of imprinted mouse genes, epigenetic memory, somatic mutation and telomeric rejuvenation of ntESCs and iPSCs have been compared [20–23]. The DNA methylation and transcriptome profiles of human ntESCs corresponds closely to *in vitro* fertilized embryonic stem cells (IVF-ESCs), whereas iPSCs exhibits differences, retaining residual DNA methylation patterns typical of parental somatic cells [24]. Comparisons of ntESCs and iPSCs can be used to identify high-quality ntESCs or iPSCs for future regenerative medicine applications. Previous studies have shown that activation of the Dlk1-Dio3 imprinted genomic region is required for TF induced iPSCs to obtain full pluripotency and the expression of the imprinted genes Rian and Gtl2 was higher in fully reprogrammed iPSCs than in partially reprogrammed iPSCs [25, 26]. However, it remains unclear whether the Dlk1-Dio3 region is also associated with ntESCs pluripotency state.

In this study, we first generated exactly syngeneic ntESCs and iPSCs from adipocyte progenitor cells (APCs) isolated from the all-iPSC mice through the primary TF mediated reprogramming in our previous study [15]. This secondary reprogramming system maintained the same genomic insertion in both ntESCs and iPSCs. By comparing fully and partially reprogrammed ntESCs and iPSCs, we observed that imprinted genes Rian and Gtl2 in the Dlk1-Dio3 region related to iPSCs pluripotency state were not correlated with the pluripotency state in ntESCs. A previous study has shown that incomplete somatic cell reprogramming caused abnormally high genomic 5-methylcytosine (5mC) levels in iPSCs compared to ntESCs and ESCs, suggesting that there might be different 5mC levels between ntESCs and iPSCs [27]. We did not observe a significant difference in 5mC or 5-hydroxymethylcytosine (5hmC) levels between fully and partially reprogrammed ntESCs and iPSCs. Our comparison of fully and partially reprogrammed ntESCs demonstrated that Grb10 was associated with the pluripotency state in ntESCs using high throughput sequencing, which was verified with quantitative reverse-transcription PCR in ntESCs from both APCs and fibroblast cells. By using syngeneic comparison, our study provides valuable information regarding ntESCs and iPSCs and identifies for the first time an important gene associated with the pluripotency state in ntESCs.

## RESULTS

### The derivation of ntESCs and iPSCs from APCs in a secondary reprogramming system

To perform an exact syngeneic comparison of ntESCs and iPSCs in this study, a secondary reprogramming system was established.

APCs isolated from the 1^0^-all-iPSC mice were used to derive ntESCs and to generate iPSCs [15, 28–31]. The 1^0^-mouse embryonic fibroblasts (MEFs)-iPSC-37 cells (37iPSC) were derived from 13.5 days postcoitum (dpc) embryos collected from female 129S2/Sv mice mated with Rosa26-M2rtTA transgenic mice and were shown to be fully pluripotency by their capacity to generate all-iPSC mice.

NtESCs were derived from the blastocysts of SCNT embryos. SCNT embryos were obtained by transferring the nuclei of APCs into enucleated oocytes (Table 1). SCNT blastocysts were plated onto a feeder layer of MEFs, and outgrowths emerged after approximately 5 to 10 days. In total, 38 ntESCs cell lines were established from 440 cloned embryos.

**Table 1:** Summary of ntESC establishment from cloned embryos with APCs

Type of donor cells	No. cloned embryos	No. morula/blastocysts (%)	No. ICM outgrowths	No. ntES cell lines(%)
APCs	187	7(3.7)	0	0(0)
APCs	131	56(42.7)	19	19(14.5)
APCs	122	54(44.3)	19	19(15.6)
Total	440	117(26.6)	38	38(8.6)

Abbreviation: APCs, adipocyte progenitor cells; ntESCs, nuclear transfer embryonic stem cells.

%: percentage of reconstructed embryos

IPSCs induced from APCs were generated by adding doxycycline. After approximately 7 days, ES-like colonies emerged. In total, 45 iPSCs cell lines were established. Hereafter, we designate the ntESCs and iPSCs from APCs using different derivation methods as AN and AI, respectively.

### Characterization of different pluripotency states in ntESCs and iPSCs derived from APCs

To evaluate the pluripotency state in syngeneic ntESCs and iPSCs, we examined the karyotypes of the derived cell lines and identified 31 AN cell lines and 41 AI cell lines with normal karyotypes (Table 2). There were only minor differences in the percentage of cell lines with a normal karyotype between the AN and AI cell lines.

**Table 2:** Summary of karyotype analysis in ntESCs and iPSCs

Type of cell lines	No. cell lines	No. normal karyotype cell lines (%)
AN	38	31(81.6)
AI	45	41(91.1)

Abbreviation: AN, nuclear transfer embryonic stem cells and adipocyte progenitor cells as donor cells; AI, induced pluripotent stem cells and adipocyte progenitor cells as primary cells.

%: percentage of normal karyotype cell lines.

**Figure 1:**
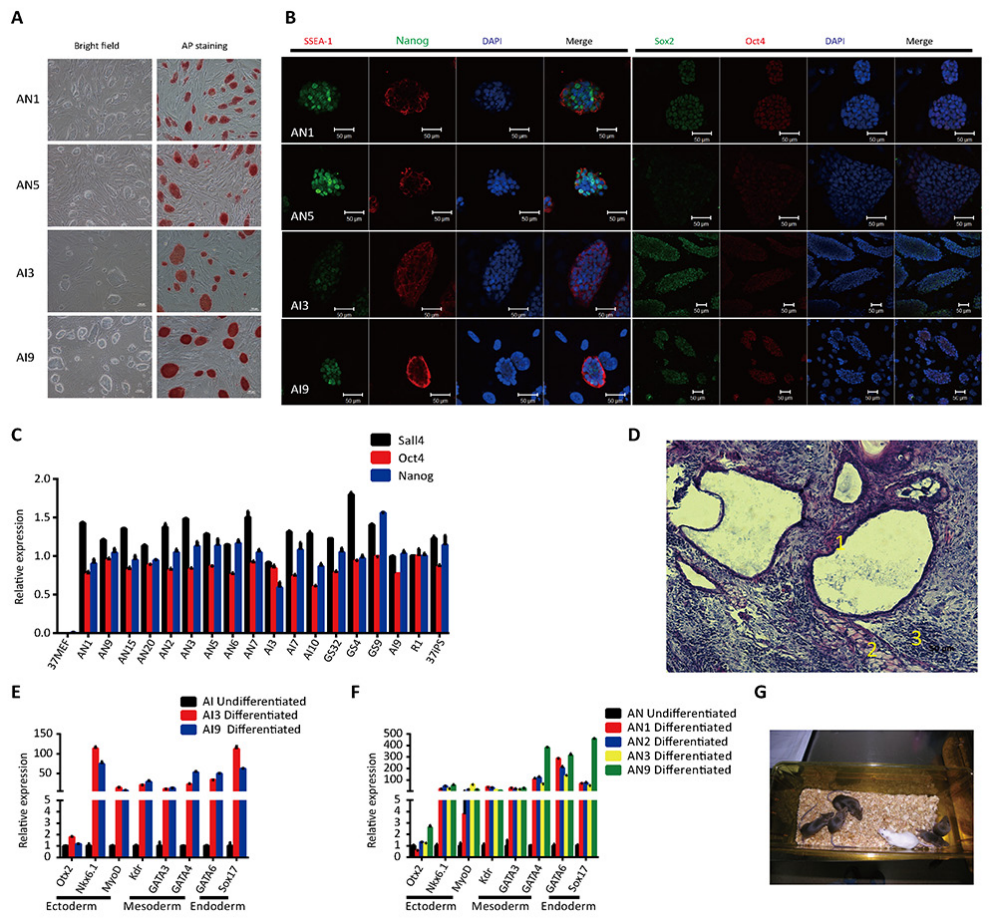
Characteristics of ntESCs and iPSCs from APCs **(A)** Morphology and alkaline phosphatase expression of ntESCs and iPSCs. Scale bars, 100 μm. **(B)** Immunofluorescence staining of pluripotent markers SSEA-1 (red), Nanog (green), Sox2 (green), and Oct4 (red) in AN and AI lines. Nuclei were stained with DAPI. Scale bars, 50μm. **(C)** Quantitative PCR analysis of the pluripotent markers Sall4, Oct4 and Nanog in AN and AI lines. Relative mRNA expression is normalized to GAPDH and is represented relative to expression in fibroblasts (37MEF). The experiments were performed in triplicate (means±SD; n=3). The primers used are listed in Supplementary Table 2. **(D)** H&E staining of teratomas generated from AN1 that formed tissues from all three germ layers. 1: Ectoderm; 2: Mesoderm; 3: Endoderm. Scale bars, 100 μm. A detailed description of all of the cell lines is given in Supplementary Table 1. **(E and F)** Quantitative PCR analysis showing the upregulation of markers for all three germ layers during the *in vitro* differentiation of AN and AI lines. Relative mRNA expression is normalized to GAPDH and represented relative to expression in undifferentiated AN and AI lines. The experiments were performed in triplicate (mean±SD; n=3). The primers used are listed in Supplementary Table 2. **(G)** Germline transmission of an AN1 tetraploid mouse. Details on all of the cell lines are in Supplementary Table 1.

Next, we observed that AN and AI cell lines exhibited a typical mouse ESCs morphology, with a compact appearance and a well-defined border. The cell lines were also positive for AP activity (Figure 1A). AN and AI cell lines expressed both protein and mRNA for pluripotency marker genes (Figure 1B and 1C).

Furthermore, we examined the developmental potential of the cell lines using teratoma formation as *in vivo* differentiation assay. Histological examination (H&E) revealed that AN and AI could give rise to teratomas containing tissues from all three germ layers (Figure 1D and Supplementary Table 1). In addition, under *in vitro* differentiation conditions, differentiated cells derived from AN and AI cell lines exhibited upregulated markers for all three germ layers compared to undifferentiated cell lines (Figure 1E and 1F).

To investigate the pluripotency state of AN and AI cell lines, we utilized a tetraploid complementation assay and performed germline transmission (Figure 1G and Supplementary Table 1). We identified four fully pluripotent ntESCs (AN1, AN9, AN15 and AN20), five partially reprogrammed ntESCs (AN2, AN3, AN5, AN6 and AN7), three fully pluripotent iPSCs (AI3, AI7 and AI10) and one partially reprogrammed iPSCs (AI9). Hereafter, we designated the fully pluripotent ntESCs as AN F, the partially reprogrammed ntESCs as AN P, the new derived fully pluripotent iPSCs and previous identified fully pluripotent GS32 as AI F, and the new derived partially reprogrammed AI9 and previous identified partially GS4 and GS9 as AI P, respectively. These fully and partially pluripotent cell lines were utilized for further investigation.

### Expression of Rian and Gtl2 in the Dlk1-Dio3 region in ntESCs and iPSCs

**Figure 2:**
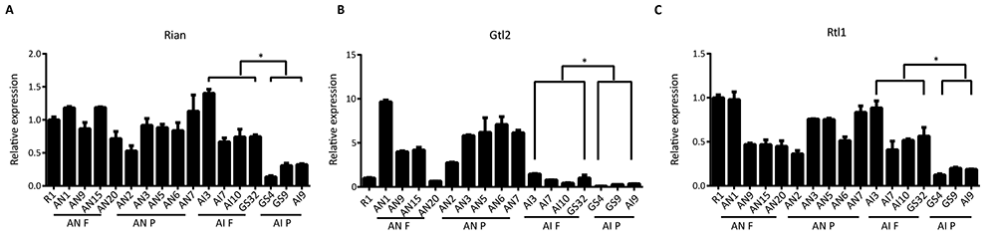
Expression of Rain, Gtl2 and Rtl1 in AN and AI cell lines **(A, B, and C)** Quantitative PCR analysis for Rain, Gtl2 and Rtl1, which are in the Dlk1-Dio3 region, in AN and AI lines. AN F: full reprogrammed cells as shown by tetraploid complementation assay in AN lines; AN P: partially reprogrammed cells as shown by tetraploid complementation assay in AN lines; AI F: full reprogrammed cells as shown by tetraploid complementation assay in AI lines; AI P: partially reprogrammed cells as shown by tetraploid complementation assay in AI lines. Relative mRNA expression is normalized to GAPDH and is represented relative to expression in R1 ESCs. The experiments were performed in triplicate (means±SD; n=3; Student's t test; *p<0.05; **p<0.01, and ***p<0.001). The primers used are listed in Supplementary Table 2.

Previous studies have shown that the imprinted genes Rian and Gtl2, which are located in the Dlk1-Dio3 region, are associated with pluripotency state in mouse iPSCs [32, 33]. The quantitative PCR results showed that the expression levels of Rian and Gtl2 were significantly different between AI F and AI P, which is consistent with previous reports showing that Rian and Gtl2 were expressed at a higher level in fully reprogrammed iPSCs than in partially reprogrammed iPSCs [25, 26]. However, we found that there was no significant difference in the expression of Rian and Gtl2 between AN F and AN P (Figure 2A and 2B), suggesting that pluripotency state related genes in the Dlk1-Dio3 region in iPSCs might not be associated with pluripotency in ntESCs. Rtl1 is another gene in the Dlk1-Dio3 region that showed little expression difference between AN F and AN P, but there was a significant difference in Rtl1 expression between AI F and AI P (Figure 2C).

### 5mC and 5hmC DNA modifications in ntESCs and iPSCs were not different

**Figure 3:**
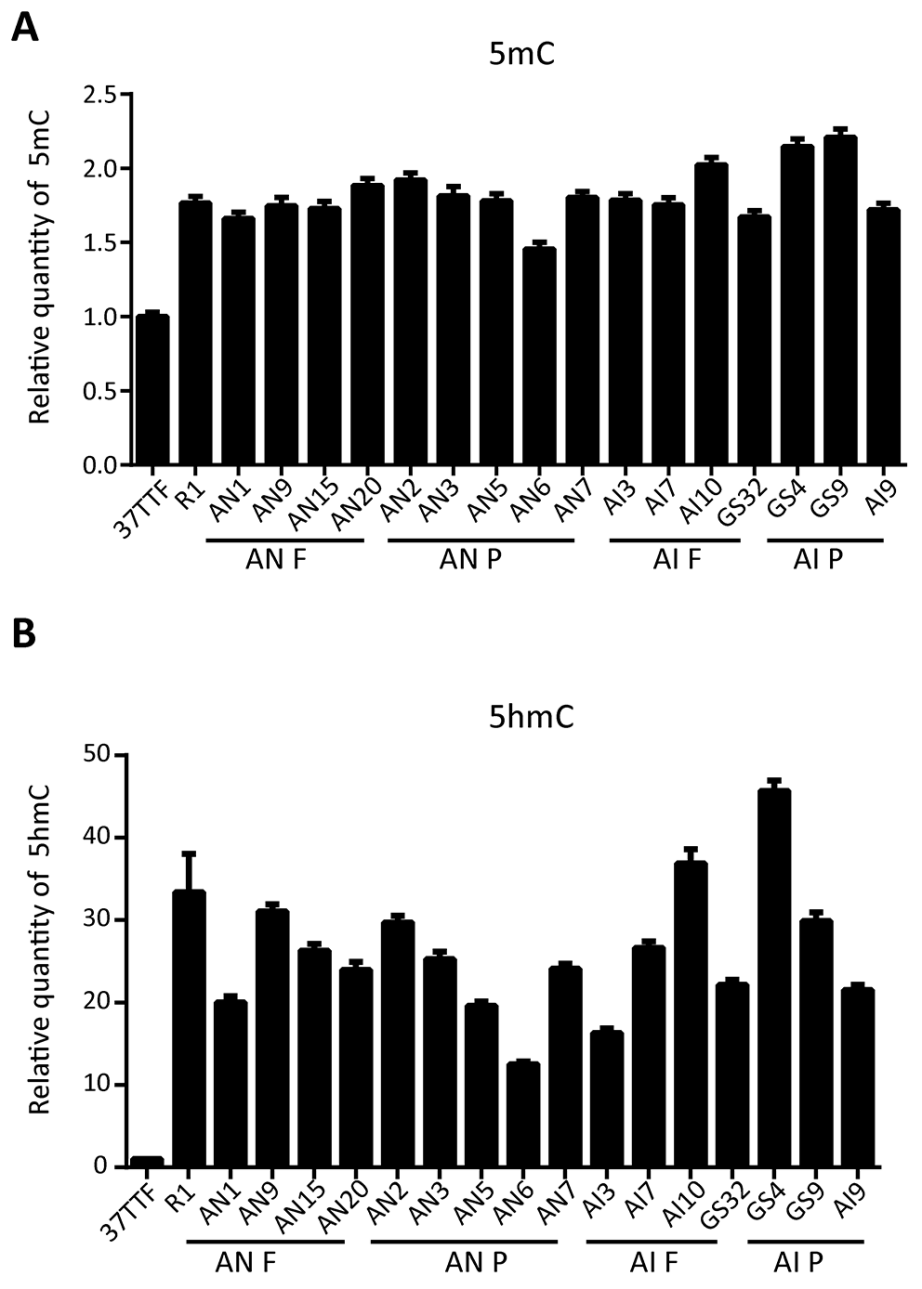
Quantification of 5mC and 5hmC in AN and AI lines **(A)** Quantification of genomic 5mC in AN F, AN P, AI F and AI P cell lines showing values that were normalized and compared to fibroblasts (37 TTF). Experiments were performed in triplicate (mean±SD; n=3). **(B)** Quantification of genomic 5hmC in AN F, AN P, AI F and AI P cell lines showing values that were normalized and compared to fibroblasts (37TTF). Experiments were performed in triplicate (mean±SD; n=3).

Evaluation of the epigenetic modification in ESCs, ntESCs and iPSCs suggested that incomplete somatic cell reprogramming might be caused by abnormally high levels of genomic 5mC in iPSC lines, even though iPSCs had germ-line chimeric properties [27]. Here, we examined the 5mC and 5hmC levels in ntESCs and iPSCs and determined whether 5mC content correlated with fully or partially reprogrammed stem cells. Our results showed that there was no significant difference in 5mC or 5hmC levels in AN F and AN P cell lines, and AI F and AI P cell lines (Figure 3A and Figure 3B).

### The identification of Grb10 associated with pluripotency state in ntESCs

**Figure 4:**
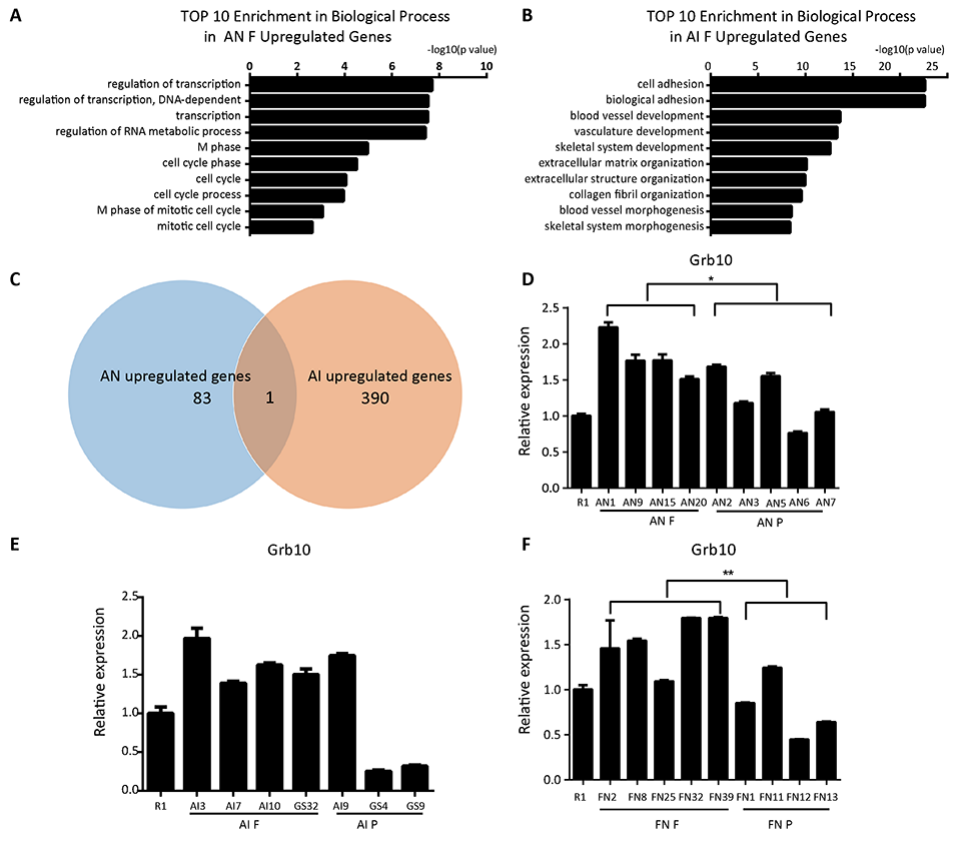
Grb10 is associated with the pluripotency state in ntESCs **(A)** GO analysis based on the upregulated genes from the comparison of fully and partially reprogrammed AN cells performed by David Bioinformatics Resources. **(B)** GO analysis based on the upregulated genes from the comparison of fully and partially reprogrammed AI cells performed by David Bioinformatics Resources. **(C)** Venn diagram representing the overlap between AI upregulated genes and AN upregulated genes. The sizes of the overlapping areas are not proportional to the actual number of genes. **(D, E and F)** Quantitative PCR analysis of Grb10 in AN, AI and FN cell lines. AN F: fully reprogrammed cells by tetraploid complementation assay in AN cell lines; AN P: partially reprogrammed cells by tetraploid complementation assay in AN cell lines; AI F: fully reprogrammed cells by tetraploid complementation assay in AI cell lines; AI P: partially reprogrammed cells by tetraploid complementation assay in AI cell lines; FN F: fully reprogrammed cells by tetraploid complementation assay in FN cell lines; FN P: partially reprogrammed cells by tetraploid complementation assay in FN cell lines. Relative mRNA expression is normalized to GAPDH and is represented relative to expression in R1 ESCs. The experiments were performed in triplicate (means±SD; n=3; Student's t test; *p<0.05; **p<0.01, and ***p<0.001). And there is no significant difference in the inside of the AIF, AIP, ANF and ANP groups (n=3, F>0.05, one-way ANOVA). The primers used are listed in Supplementary Table 2.

We used high throughput sequencing to compare gene expression differences between AN F and AN P, and AI F and AI P. In AN F, 84 genes were significantly upregulated, and 391 genes were upregulated in AI F compared to AN P and AI P, respectively. Gene Ontology (GO) analysis showed that most of the upregulated genes in AN F were related to transcription (Figure 4A). The upregulated genes in AI F were enriched for mesodermal development-related processes including blood vessel, vasculature and skeletal development (Figure 4B). Interestingly, we found that Grb10, a maternally imprinted gene, was upregulated in both the AI F and AN F cell lines (Figure 4C). Our quantitative PCR analysis confirmed that Grb10 expression in fully reprogrammed AN cells was higher than in partially reprogrammed cells (Figure 4D). In AI cell lines, Grb10 expression in most AI fully reprogrammed cell lines was higher than in partially reprogrammed cell lines except for AI9 (Figure 4E). These results suggest that Grb10 might be associated with pluripotency state in ntESCs. To further verify that Grb10 was associated with pluripotency state in ntESCs, ntESCs cells (FN) derived from SCNT blastocysts using the tail-tip fibroblasts (TTFs) as donor cells were examined. The TTFs were from the 1^0^-all-iPSC mice in the primary TF induced pluripotent reprogramming in our previous study [15]. The 1^0^-MEF-iPSC-37 cells (37iPSC) were derived from 13.5 dpc embryos collected from female 129S2/Sv mice mated with Rosa26-M2rtTA transgenic mice and were shown to be fully pluripotency by their capacity to generate the all-iPSC mice. FN cell lines were also grouped into fully and partially reprogrammed cell lines using the tetraploid complementation assay and were designated FN F and FN P, respectively (unpublished data). This assay showed that Grb10 expression was significantly higher in FN F than in FN P (Figure 4F), which indicates that Grb10 might work as an important molecular marker for indicating pluripotency state in ntESCs derived from different cell types.

## DISCUSSION

In this study, we compared syngeneic ntESCs and iPSCs and showed that the expression of pluripotency associated genes in Dlk1-Dio3 region and 5mC/5hmC levels could not be used to evaluate fully and partial reprogrammed ntESCs, and demonstrated that Grb10 is associated with the pluripotency state in ntESCs.

Imprinted genes are expressed from a single parental allele and have parental-specific epigenetic modifications [34]. The imprinted genes Rian and Gtl2 are located in Dlk1-Dio3 region on distal mouse chromosome 12 [32, 33]. A previous study indicated that Rian and Gtl2 are significantly downregulated transcripts in iPSCs when comparing genome wide expression between genetically identical mouse ESCs and iPSCs [25]. In our study, the expression of Rian, Gtl2 and Rtl1 was higher in AI F than in AI P and was associated with iPSCs quality, which was consistent with the previous studies [25, 26]. It remains unclear that whether the expression of Rian, Gtl2 and Rtl1 are also associated with the pluripotency state in ntESCs. To evaluate the pluripotency state in ntESCs, we examined these associated genes in more AN cell lines in this study. There was little difference in the expression of Rian, Gtl2, and Rtl1 between AN F and AN P, which indicated that Rian, Gtl2 and Rtl1 expression from the Dlk1-Dio3 region is not useful to evaluate fully and partial reprogrammed ntESCs. A previous study indicated that TF stoichiometry influences the Dlk1-Dio3 locus and that some Gtl2-LOW (not OFF) iPSCs can support the iPSC full-term development [35]. Gtl2 might activate the Dlk1-Dio3 region and be used to assess the quality of reprogramming in iPSCs [36]. It showed that the expression of Gtl2 was associated to the quality of iPSCs. Our results showed that Gtl2 expression in AN cell lines was not associated with the pluripotency state in ntESCs and Gtl2 could not be used to assess the quality of reprogramming in ntESCs.

Epigenetic modifications include DNA methylation and histone modification, which play important roles in both SCNT reprogramming and TF induced pluripotent reprogramming. Cloning of mammals by SCNT results in gestational or neonatal failure with at most a few percent of manipulated embryos resulting in live births likely due to the inappropriate epigenetic reprogramming [35]. The identity of somatic cells is strictly protected by an epigenetic barrier, and these cells acquire pluripotency by breaking the epigenetic barrier by reprogramming factors such as Oct3/4, Sox2, Klf4, Myc and LIN28 [37, 38]. A previous report indicates that a major reprogramming event during early embryonic development is the erasure and subsequent re-establishment of methylation patterns at 5mC [39]. 5hmC is an epigenetic modification that has been suggested to be associated with the pluripotency state during reprogramming of mouse fibroblasts into iPSCs [40]. Our previous results suggested that 5mC-to-5hmC conversion represents a crucial step in the initiation of epigenetic remodeling and transcriptome resetting to achieve pluripotency [41]. A previous report found that the genomic 5mC levels in iPSCs were higher than the levels in ntESCs [27]. Therefore, we compared the levels of 5mC and 5hmC in both ntESCs and iPSCs. However, we found no difference in 5mC and 5hmC levels between the AN F and AN P cell lines, or the AI F and AI P cell lines. This result was not consistent with previous study that reported ntESCs and iPSCs have different 5mC levels. In this study, we used nine ntESC cell lines and seven iPSC cell lines, which were syngeneic with same genomic insertion, to examine 5mC and 5hmC levels. These stem cells were well defined as fully and partially reprogrammed ntESCs and iPSCs by tetraploid complementation assay. We used more cell lines than the previous study, which used only two ntESCs and two germ-line chimeric iPSCs without further performing tetraploid complementation assay. Our results suggest that genomic 5mC levels might not be used to evaluate pluripotency state in AN and AI cell lines and that the dynamic conversion of 5mC to 5hmC might become stable after reprogramming is complete.

To evaluate the pluripotency state of AN cell lines, a comparison of fully and partially reprogrammed cell lines was performed using high throughput sequencing. To avoid the difference caused by RNA library construction, quality control, quantification and other sequencing processes, we performed high throughput sequencing in all AN and AI cell lines together, including previous identified AI cell lines. Interestingly, our result showed a significant difference between AN F and AN P in the expression levels of Grb10 (Figure 4D), which suggested that Grb10 might be a marker for the pluripotency state in ntESCs. Grb10, which is also called Meg1, is an imprinted gene on mouse proximal chromosome 11 and a candidate gene causing Silver-Russell syndrome [42]. Previous reports suggested that aberrant function of Grb10 may contribute to disorders of proliferation, apoptosis, and metabolism, with specific emphasis on growth and neuronal development [43–46]. Recent study has indicated that Grb10 plays an inhibitory role for hematopoietic stem cell self-renewal and regeneration [47]. We examined Grb10 expression in another series of fully and partially reprogrammed ntESCs derived from fibroblasts and verified that Grb10 is associated with the pluripotency state in ntESCs in our study, although the mechanism for this association remains unclear.

In summary, our study performed an invaluable comparison of syngeneic ntESCs and iPSCs and identified for the first time that an imprinted gene, Grb10, is associated with the pluripotency state in ntESCs.

## MATERIALS AND METHODS

### Mice and cell culture

All of the animal protocols and experiments were approved by the Animal Research Committee of the Institute of Zoology, Chinese Academy of Sciences, and are consistent with the National Institute of Biological Sciences guide for the care and use of laboratory animals.

Female B6D2F1 (C57BL/6XDBA/2) mice (8-10 weeks old) were superovulated by sequential injection with 7 IU of pregnant mare serum gonadotropin (PMSG) and human chorionic gonadotropin (hCG) (San-Sheng Pharmaceutical Co. Ltd, Ningbo, China). Metaphase II (MII) oocytes were collected for SCNT experiments as previously described [48].

MEFs were derived from 13.5 dpc embryos collected from ICR mice and were cultured in Fundamental Culture medium (FM) containing Dulbecco's modified Eagle's medium (DMEM) (Invitrogen, Thermo Fisher Scientific, Waltham, MA, USA) supplemented with 10% (v/v) fetal bovine serum (FBS) (Invitrogen, Thermo Fisher Scientific) and 1 mM L-glutamine (Merck, Millipore, Billerica, MA, USA). ESCs culture medium contains DMEM (Merck, Millipore) supplemented with 15% (v/v) FBS (Hyclone, Logan, Utah), 1 mM L-glutamine (Merck, Millipore), 0.1 mM mercaptoethanol (Merck, Millipore), 1% non-essential amino acid stock (Merck, Millipore), penicillin/streptomycin (Merck, Millipore), nucleosides (Merck, Millipore), and 1,000 U/ml Leukemia Inhibitory Factor (LIF) (Merck, Millipore).

Adipocyte progenitor cells (1^0^-APCs), 37 tail-tip fibroblasts (1^0^-TTFs) and 37 MEFs (1^0^-MEFs) were isolated from 1^0^-all-iPSC mice produced by 1^0^-MEF-iPSC-37 cells, which were shown to be fully pluripotent by their capacity to generate all-iPSC mice [15].

AN cell lines were derived from SCNT blastocysts using 1^0^-APCs as donor cells. AI cell lines were generated from 1^0^-APCs by adding doxycycline to the induction medium. Previously well-defined 2^0^-APC-iPSC-4, 2^0^-APC-iPSC-9, and 2^0^-APC-iPSC-32 cell lines were named GS4, GS9, and GS32, respectively, for these experiments [28–31].

FN cell lines were derived from SCNT blastocysts using 1^0^-TTFs as the donor cells.

A schematic of the cell lines derivation is shown in Supplementary Figure 1.

### Alkaline phosphatase (AP) staining

AP staining was performed with a Leukocyte Alkaline Phosphatase Kit (Sigma, St Louis, MO, USA) according to the manufacturer's instructions.

### Karyotype analysis

The cells were incubated in ESCs medium with 0.25 μg/ml colcemid (Invitrogen, Thermo Fisher Scientific) for 2-3 h and harvested with 0.05% Trypsin-EDTA (Invitrogen, Thermo Fisher Scientific). After incubation in hypotonic solution containing 0.4% sodium citrate and 0.4% potassium chloride (1:1, v/v) at 37°C for 5 min, the cells were fixed with a methanol/acetic acid mixture (3:1, v/v). The fixed cells were mounted on glass slides and stained with Giemsa at 37°C for 10-15 min after drying. At least 20 metaphase chromosome karyoschisis were examined for each cell line.

### Immunofluorescence staining

For immunofluorescence staining, AN and AI cells were seeded on gelatin-coated cover slips and fixed with 4% paraformaldehyde. After permeabilization with 0.5% Triton-X and blocking with 0.5% bovine serum albumin (BSA), the cells were incubated with primary antibodies against Oct4 (1:500, Santa Cruz, Dallas, TX, USA), Sox2 (1:500, Santa Cruz), Nanog (1:500, COSMO BioCo, Tokyo, Japan) and SSEA-1 (1:50, Merck, Millipore). Then, the cells were incubated with the appropriate secondary antibodies after washing three times. DNA was labeled with DAPI (Merck, Millipore). Stained cells were mounted on cover slips and observed using a LSM 510 META microscope (Zeiss, North York, ON, Canada).

### Teratoma formation

AN and AI cells (2-5×10^6^) were subcutaneously injected into the groin of severe combined immune deficiency (SCID) mice. Tumors were dissected and processed for hematoxylin-eosin staining 6-8 weeks after injection.

### Embryoid body formation

AN and AI cells were trypsinized into a single cell suspension and transferred to Petri dishes in DMEM supplemented with 15% FBS without LIF. Three to seven days later, the embryoid bodies (EBs) were harvested and plated onto gelatin-coated tissue culture dishes for another 3-7 days. Total RNA from plated EBs was extracted and used for quantitative PCR. GAPDH was used as an endogenous control.

### Quantitative reverse-transcription PCR

Total RNA was purified using TRIzol (Invitrogen, Thermo Fisher Scientific). RNA (2 μg) was reverse-transcribed using M-MLV Reverse Transcriptase and RNasin RNase Inhibitor (Promega, Madison, WI, USA). Quantitative reverse-transcription PCR was performed using SYBR Premix Ex Taq (Takara, Kusatsu, Japan). The reactions were performed in triplicate on a 1/10 dilution of the cDNA obtained from above. Gene expression in each sample was normalized to GAPDH, and the relative quantification of expression was estimated using the comparative CT method. All of the primers used are listed in Supplementary Table 2.

### Tetraploid complementation

To perform tetraploid complementation, B6D2F1 embryos at the 2-cell stage were electrofused to tetraploid embryos, 10-15 cells were injected into the reconstructed tetraploid blastocysts, and these were transplanted into the uteri of pseudo-pregnant mice. Caesarean sections were performed on day 19.5, and pups were fostered by lactating ICR mothers.

### RNA-seq data analysis

Total RNA was extracted from the different cell lines using TRIzol (Invitrogen, Thermo Fisher Scientific). Libraries were constructed with the NEBNext DNA Library Prep Master Mix Set for Illumina (New England Biolabs, MA, USA), and PCR products were purified using AMPure XP beads (Beckman, Brea, CA, USA). The RNA library was quantified using Qubit 1.0 (Invitrogen, Thermo Fisher Scientific), analyzed using an Agilent 2100 Bioanalyzer (Agilent Technologies, Santa Clara, CA, USA) for size distribution, and then sequenced with an Illumina Hiseq-2500 in single mode (1×50nt) by the Bioinformatics core facility at National institute of Biological Sciences, Beijing.

The 51 bp sequences called by the Illumina pipeline were mapped to the mouse genome (mm9) using Tophat (v2.1.0) for data analysis. Gene annotation and calculation of FPKM values was performed using Cufflinks (v2.2.1) with the GTF annotation file (mm9).

Gene expression differences were assessed by Cuffdiff with a false discovery rate correction for multiple testing. Genes with a p-value < 0.05 and q-value < 0.05 were considered differentially expressed.

To identify overrepresented biological categories within each cluster, GO analysis was applied (DAVID, https://david.ncifcrf.gov/).

### Liquid chromatography-mass spectrometry (LC-MS/MS) Analysis of 5mC and 5hmC

The genomic DNA (5 μg) from different cell lines was analyzed by liquid chromatography-tandem mass spectrometry to assess the quantity of 5mC and 5hmC, as described in our previous study [41, 49, 50].

### Accession numbers

All the high-throughput sequencing data sets can be accessed as the GEO reference GSE92308 (https://www.ncbi.nlm.nih.gov/geo/query/acc.cgi?token=ezmjmmkmpfcxngv&acc=GSE92308).

### Statistics

Student's t tests were performed using SigmaStat 3.5 software for statistical comparisons. And one-way ANOVA were performed using SPSS Statistics 19 software.

## SUPPLEMENTARY MATERIALS FIGURES AND TABLES





## Abbreviations

5hmC: 5-hydroxymethylcytosine
5mC: 5-methylcytosine
AI F: fully reprogrammed cells by tetraploid complementation assay in AI cell lines
AI P: partially reprogrammed cells by tetraploid complementation assay in AI cell lines
AI: induced pluripotent stem cells and adipocyte progenitor cells as primary cells
AN F: fully reprogrammed cells by tetraploid complementation assay in AN cell lines
AN P: partially reprogrammed cells by tetraploid complementation assay in AN cell lines
AN: nuclear transfer embryonic stem cells and adipocyte progenitor cells as donor cells
AP Staining: Alkaline Phosphatase staining
APCs/1^0^-APCs: adipocyte progenitor cells
B6D2F1 mice: C57BL/6XDBA/2 mice
BSA: bovine serum albumin
DMEM: Dulbecco's modified Eagle's medium
dpc: days postcoitum
EBs: Embryoid bodies
ESC: embryonic stem cell
FBS: fetal bovine serum
FM: Fundamental Culture medium
FN F: fully reprogrammed cells by tetraploid complementation assay in FN cell lines
FN P: partially reprogrammed cells by tetraploid complementation assay in FN cell lines
FN: nuclear transfer embryonic stem cells and tail-tip fibroblasts as donor cells
GO analysis: Gene Ontology analysis
GS32: 2^0^-APC-iPSC-32 cell line
GS4: 2^0^-APC-iPSC-4 cell line
GS9: 2^0^-APC-iPSC-9 cell line
H&E: Histological examination
hCG: human chorionic gonadotropin
iPSCs: induced pluripotent stem cells
IVF-ESCs: *in vitro* fertilized embryonic stem cells
LC-MS/MS: Liquid Chromatography-Mass Spectro-metry.
LIF: Leukemia Inhibitory Factor
MEFs: mouse embryonic fibroblasts
MII: Metaphase II
ntESCs: nuclear transfer embryonic stem cells
PMSG: pregnant mare serum gonadotropin
SCID mice: severe combined immune deficiency mice
SCNT: somatic cell nuclear transfer
SCR: scriptaid
TF: transcription factor
TSA: trichostatin A
TTFs /1^0^-TTFs: tail-tip fibroblasts;
