# Supplementary material for: High throughput sequencing identifies an imprinted gene, Grb10, associated with the pluripotency state in nuclear transfer embryonic stem cells
Source: Oncotarget. 2017 Apr 18;8(29):47344–55. doi: 10.18632/oncotarget.17185 (PMC5564569; doi:10.18632/oncotarget.17185)
Supplement: Supplementary file 1 [file oncotarget-08-47344-s001.pdf]

High throughput sequencing identifies an imprinted gene, Grb10, associated with the pluripotency state in nuclear transfer embryonic stem cells

SUPPLEMENTARY MATERIALS

SUPPLEMENTARY FIGURE AND TABLES

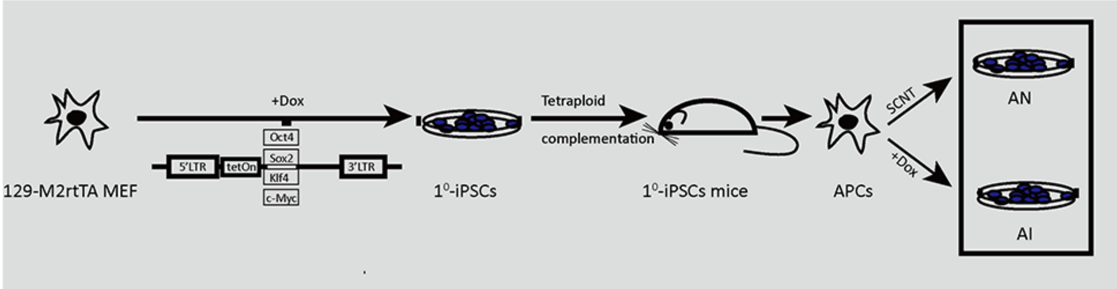

Supplementary Figure 1: A schematic diagram for establishment of ntESCs and iPSCs from APCs. A schematic diagram for the production of ntESCs and iPSCs using APCs from tetraploid mice by primary TF mediated induced pluripotent reprogramming.

Supplementary Table 1: Summary of generation of teratomas and tetraploid complementation assay

Cell lines	Teratomas	*In vivo* differentiation	Blastocysts injected	No. cells injected	Pups born(adult)	Germline transmission
AN1	Yes	Yes	710	12-15	23(5)	Yes
AN9	Yes	Yes	270	12-15	17(0)	No data
AN15	Yes	Yes	70	12-15	3(0)	No data
AN20	Yes	Yes	70	12-15	3(0)	No data
AN2	Yes	Yes	300	12-15	0(0)	--
AN3	Yes	Yes	330	12-15	0(0)	--
AN5	Yes	Yes	320	12-15	0(0)	--
AN6	Yes	Yes	370	12-15	0(0)	--
AN7	Yes	Yes	420	12-15	0(0)	--
AI3	Yes	Yes	40	12-15	5(1)	Yes
AI7	Yes	Yes	160	12-15	5(5)	Yes
AI10	Yes	Yes	220	12-15	6(0)	No data
AI9	Yes	Yes	160	12-15	0(0)	--

Abbreviation: AN, nuclear transfer embryonic stem cells and adipocyte progenitor cells as donor cells; AI, induced pluripotent stem cells and adipocyte progenitor cells as primary cells.
Note: No data indicates there are no adult tetraploid mice for germline transmission; "--"indicates there are no tetraploid mice.

Supplementary Table 2: Primer sequences.

See Supplementary File 1
