# Supplementary material for: High throughput sequencing identifies an imprinted gene, Grb10, associated with the pluripotency state in nuclear transfer embryonic stem cells
Source: Oncotarget. 2017 Apr 18;8(29):47344–55. doi: 10.18632/oncotarget.17185 (PMC5564569; doi:10.18632/oncotarget.17185)
Supplement: Supplementary file 2 [file oncotarget-08-47344-s002.docx]

Table S2 Primer sequences

| Locus Name | Primer sequences |
| --- | --- |
| Markers for three germ layers |  |
| Otx2 | Forward- TATCTAAAGCAACCGCCTTACG |
|  | Reverse- AAGTCCATACCCGAAGTGGTC |
| Nkx6.1 | Forward-CAGACCCACGTTCTCTGGAC |
|  | Reverse-TGCGTGCTTCTTTCTCCACT |
| MyoD | Forward-CCACTCCGGGACATAGACTTG |
|  | Reverse-AAAAGCGCAGGTCTGGTGAG |
| Kdr | Forward-TTTGGCAAATACAACCCTTCAGA |
|  | Reverse-GCAGAAGATACTGTCACCACC |
| GATA3 | Forward- CATTACCACCTATCCGCCCTATG |
|  | Reverse- CACACACTCCCTGCCTTCTGT |
| GATA4 | Forward-CCCTACCCAGCCTACATGG |
|  | Reverse-ACATATCGAGATTGGGGTGTCT |
| GATA6 | Forward-TTGCTCCGGTAACAGCAGTG |
|  | Reverse-GTGGTCGCTTGTGTAGAAGGA |
| Sox17 | Forward-GATGCGGGATACGCCAGTG |
|  | Reverse-CCACCTCGCCTTTCACCTTTA |
| Markers for pluripotency |  |
| Sall4 | Forward-GTTAGATGTCAAGGCCAAGGAC |
|  | Reverse-GGCGTCTACAGAGAGACTCGAT |
| Oct4 | Forward-ACCTGGCTTCAGACTTCGC |
|  | Reverse-TGAGCCTGGTCCGATTCCA |
| Nanog | Forward-TCTTCCTGGTCCCCACAGTTT |
|  | Reverse-GCAAGAATAGTTCTCGGGATGAA |
| RNA-seq analysis |  |
| Rian | Forward-ATGCGATCTGAGTACTGAGAGG |
|  | Reverse-AAGGCTGAAGGAGCTGAAGG |
| Rtl1 | Forward-CCTGTGCCAGGGGCTCAACG |
|  | Reverse-CTTGGGCGCGACTCAGGTGG |
| Gtl2 | Forward-TTGCACATTTCCTGTGGGAC |
|  | Reverse-AAGCACCATGAGCCACTAGG |
| Grb10 | Forward- TGCACCACTTCTTGAGGATG |
|  | Reverse- ACCAGTGAGCTCCGGAAATG |
